# Regulation of mTOR complexes in long-lived growth hormone receptor knockout and Snell dwarf mice

**DOI:** 10.18632/aging.203959

**Published:** 2022-03-19

**Authors:** Xiaofang Shi, S. Joseph Endicott, Richard A. Miller

**Affiliations:** 1 Department of Pathology, University of Michigan Medical School, Ann Arbor, MI 48109, USA; 2 University of Michigan Geriatrics Center, Ann Arbor, MI 48109, USA; 3 Paul F. Glenn Center for Biology of Aging Research, University of Michigan Medical School, Ann Arbor, MI 48109, USA

**Keywords:** aging, lifespan extension, mTOR, TSC, growth hormone receptor

## Abstract

Downregulation of mTOR (mechanistic target of rapamycin) can extend lifespan in multiple species, including mice. Growth hormone receptor knockout mice (GHRKO) and Snell dwarf mice have 40% or greater lifespan increase, and have lower mTORC1 function, which might reflect alteration in mTORC1 components or alteration of upstream proteins that modulate mTOR activity. Here we report reduction of mTORC components DEPTOR and PRAS40 in liver of these long-lived mice; these changes are opposite in direction to those that would be expected to lead to lower mTORC1 function. In contrast, levels of the upstream regulators TSC1 and TSC2 are elevated in GHRKO and Snell liver, kidney and skeletal muscle, and the ratio of phosphorylated TSC2 to total TSC2 is lower in the tissues of the long-lived mutant mice. In addition, knocking down TSC2 in GHRKO fibroblasts reversed the effects of the GHRKO mutation on mTORC1 function. Thus increased amounts of unphosphorylated, active, inhibitory TSC may contribute to lower mTORC1 function in these mice.

## INTRODUCTION

**Figure 1:**
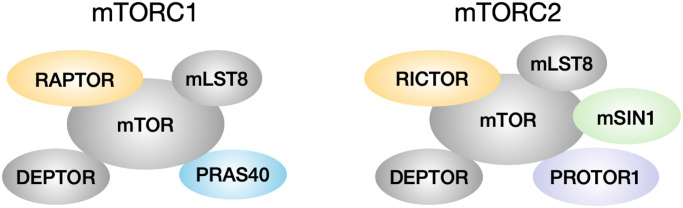
**mTORC1 and mTORC2 composition.** mTORC1 has core components mTOR, mLST8 and RAPTOR, and is associated with inhibitory regulators DEPTOR and PRAS40. mTORC2 has core components mTOR, mLST8, RICTOR and mSIN1, and is associated with inhibitory regulator DEPTOR and positive regulator PROTOR1.

Lower levels of mTOR function are associated with longer lifespan in many species. mTOR deficiency in yeast, worms and flies leads to lifespan extension in each of these species [1–3]. Mice given the mTOR inhibitor rapamycin also have their lifespan increased by as much as 23% in males and 26% in females at the highest dose tested [4]. mTOR in mammals can interact with other components to form two complexes, mTORC1 and mTORC2 (Figure 1). mTORC1 consists of the core kinase mTOR, the scaffold protein RAPTOR, the stabilizer mLST8, and inhibitory proteins DEPTOR and PRAS40 [5–8]. mTORC2 contains the mTOR kinase, the scaffold protein RICTOR, mLST8, DEPTOR, an indispensable subunit mSIN1, and the positive regulator PROTOR1 [9–11]. Among these proteins, mTOR, mLST8, RAPTOR, RICTOR and mSIN1 are core components of the complexes, whereas DEPTOR, PRAS40 and PROTOR1 are considered as associated regulatory components [12]. mTORC1 function is influenced by multiple signals including growth factors insulin, EGF, and IGF-1, amino acids, and ATP level, to regulate protein synthesis, autophagy, cell growth and metabolism [12]. Upon activation, mTOR1 phosphorylates downstream substrates such as S6K and 4EBP1, to control ribosome biogenesis and protein synthesis [13, 14]. mTORC2 responds to insulin signaling and is involved in apoptosis and cell migration [15]. mTORC2 phosphorylates substrates, for example, AKT on serine 473 and threonine 450, to regulate cell survival and proliferation [16].

The insulin/IGF-1 signaling pathway is a critical focus in aging regulation. Originally, it was reported that *C. elegans* with mutations in the insulin/IGF-1 receptor *daf-2* have doubled lifespan [17]. Since then, several mouse models have also been developed to show the lifespan extension effect by manipulating genes involved in the GH/IGF-1 signaling. For example, GHRKO mice with disruption of their growth hormone receptor live about 40–50% longer than wildtype controls [18]. Snell dwarf mice with the Pit1 (Pituitary-specific positive transcription factor 1) gene mutation have reduced serum growth hormone and IGF-1 levels, and live more than 40% longer than littermate controls [19]. It was previously reported [20] that liver, kidney, muscle and heart of these GHRKO and Snell dwarf mice have lower mTORC1 activity shown by lower S6K and 4EBP1 phosphorylation. Interestingly, phosphorylation of several but not all mTORC2 substrates is higher in these tissues of GHRKO and Snell dwarf mice under fasted conditions, suggesting higher mTORC2 activity with differential regulation of mTORC2 targets. However, it is unknown what is regulating the mTORC activity or substrate specificity here.

mTORC1 function is modulated by growth factors, amino acids, and energy levels. These signals converge on an upstream regulator TSC [12], a complex composed of TSC1, TSC2 and TBC1D7. TSC2 inhibits mTORC1 activity through the mTORC1 activator Rheb [21]. TSC1 stabilizes TSC2 by blocking its ubiquitination [22]. TBC1D7 promotes the association of TSC1 and TSC2 [23]. It is not clear whether TSC is involved in the regulation of lower mTORC1 function in the slow-aging GHRKO or Snell dwarf mice.

To answer these questions, we tested the levels of various mTORC components in GHRKO and Snell dwarf mouse tissues to see whether these proteins are differentially regulated in these mice. We found unexpected alternation in two components DEPTOR and PRAS40, and further study by knocking down these two genes did not change the phosphorylation of mTORC substrates. We also tested the levels and phosphorylation status of TSC components, and found upregulated TSC signaling in these mice. Furthermore, knocking down TSC2 in GHRKO cells led to increased phosphorylation of mTORC1 substrates. These results suggest that the lower mTORC1 activity might be regulated by higher TSC signals in the long-lived GHRKO and Snell dwarf mice.

## RESULTS

### Protein expression of mTORC components in GHRKO tissues

**Figure 2:**
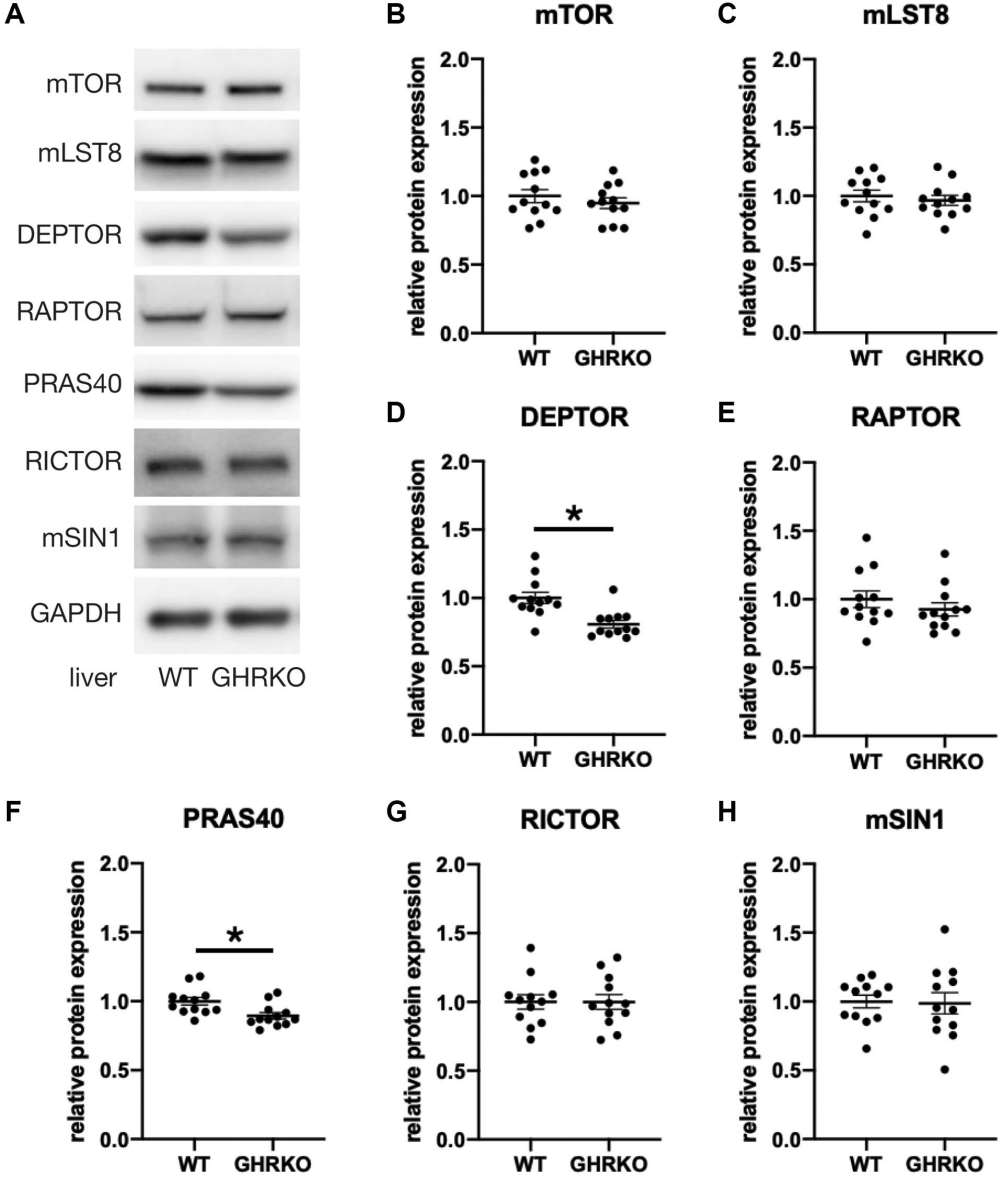
**Reduced DEPTOR and PRAS40 protein expression in GHRKO liver.** (**A**) Representative immunoblots of protein expression for mTORC1 and mTORC2 components. (**B**–**H**) Quantification of protein expression, for *N* = 6 male and *N* = 6 female mice, with mean and SEM. ^*^t-test *p*-value < 0.05.

To study the protein expression of mTORC components in GHRKO mice, we extracted protein samples from liver, kidney and skeletal muscle, and prepared immunoblots. Figure 2 shows representative images and statistical quantification for liver tissue, using six mice of each sex for each genotype. Since two-way ANOVA found no effects of sex and no (sex × genotype) interactions for any of these proteins, we pooled the data for males and females for our analyses. We found lower levels of DEPTOR and PRAS40 in GHRKO liver compared with wildtype mice. The other components were not altered between control and GHRKO mice, including the shared components mTOR and mLST8, the mTORC1 specific component RAPTOR, and the mTORC2 specific components RICTOR and mSIN1. DEPTOR is present in both mTORC1 and mTORC2, and inhibits their activity [7]. PRAS40 is present in, and inhibits, mTORC1 [8]. Since each of these inhibitory mTORC components decreases in GHRKO liver, these changes are unlikely to explain the decrease in mTORC1 function seen in liver of GHRKO mice [20]. In kidney, expression of DEPTOR and PRAS40 did not differ between wildtype and GHRKO mice (Supplementary Figure 1A). Muscle of GHRKO mice showed increased DEPTOR but unchanged PRAS40 (Supplementary Figure 1B). Thus the effects of lifespan-extending mutations on levels of mTORC components are not consistent among different tissues.

To check the generality of these findings, we used Snell dwarf mice to evaluate genetic effects on mTORC components. Consistent with the findings in GHRKO mice, we again saw reduced levels of DEPTOR and PRAS40 in the liver tissue but unaltered expression of the other mTORC components (Supplementary Figure 2). Similar to GHRKO kidney, Snell dwarf kidney showed no difference in DEPTOR and PRAS40 expression. PRAS40 but not DEPTOR was decreased in Snell dwarf muscle (Supplementary Figure 3). Thus genetic effects on mTORC components are inconsistent in muscle, not apparent in kidney, and consistent in liver but in a direction that does not explain lower mTORC1 function in these long-lived mice.

### Protein expression of mTORC components in immunoprecipitates

**Figure 3:**
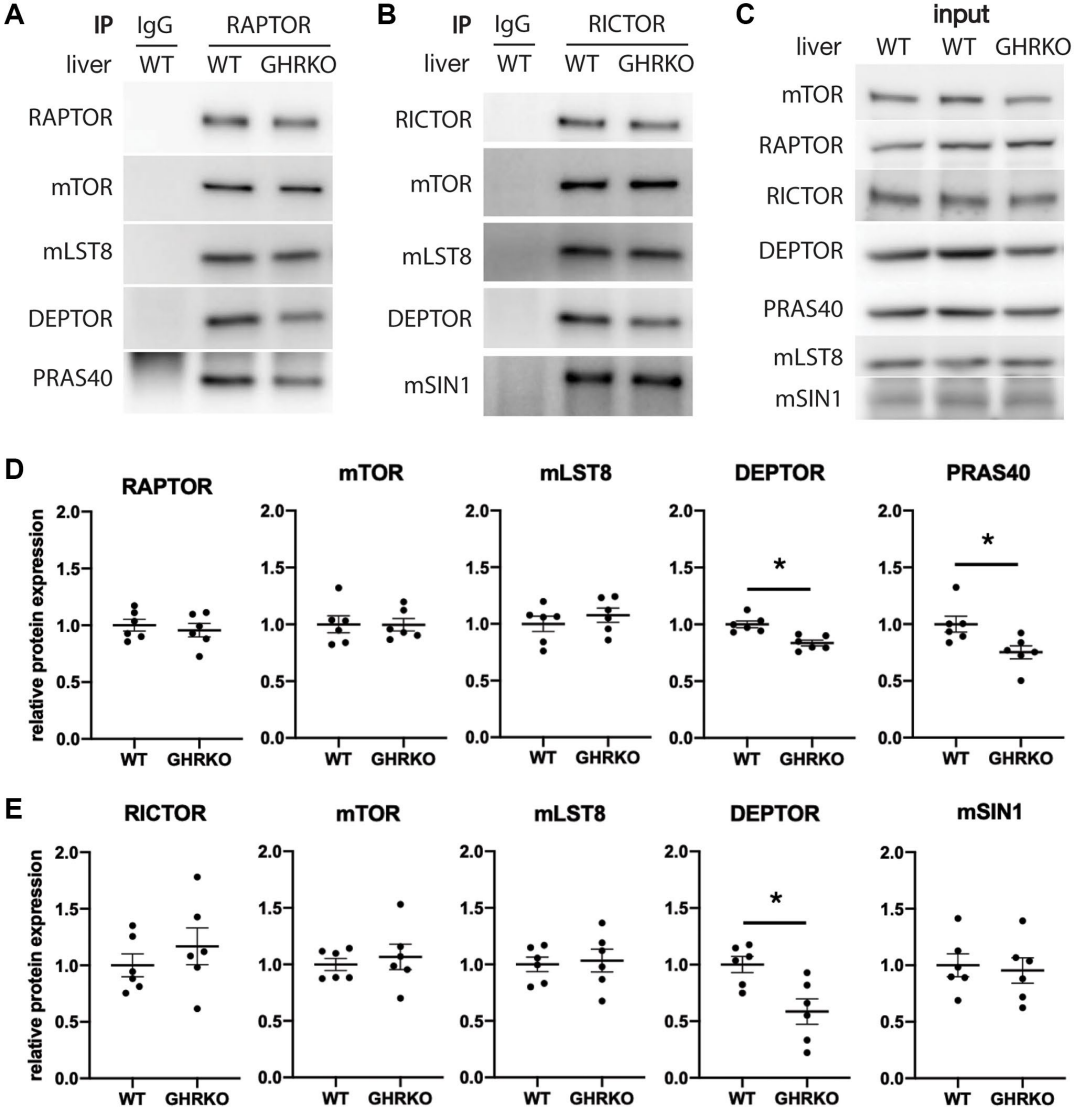
**Decreased DEPTOR and PRAS40 in immunoprecipitated mTOR complexes.** (**A**) Representative immunoblots of the mTORC1 components from samples immunoprecipitated with anti-RAPTOR antibody. (**B**) Representative immunoblots of the mTORC2 components immunoprecipitated with anti-RICTOR antibody. (**C**) Representative immunoblots of the input samples. (**D**) Quantification of the protein levels in RAPTOR-IP samples, as in (**A**). (**E**) Quantification of the protein levels in RICTOR-IP samples, as in (**B**), for *N* = 6 male mice, with mean and SEM. ^*^t-test *p* < 0.05.

To estimate levels of each component within the mTOR complexes, we carried out immunoprecipitation experiments using antibodies specific for the scaffold protein RAPTOR to pull down mTORC1, and using anti-RICTOR antibody to pull down mTORC2. We measured levels of the mTORC1 components mTOR, mLST8, DEPTOR and PRAS40 in the RAPTOR IP samples from control and GHRKO liver, and found lower levels of DEPTOR and PRAS40 in mTORC1 immunoprecipitated from GHRKO compared to controls, which is consistent with the findings from whole liver lysates (Figure 3A, 3D). RICTOR IP samples from GHRKO mice contained less DEPTOR but had unaltered expression of the other components mTOR, mLST8, and mSIN1 (Figure 3B, 3E). This suggests that not only are levels of DEPTOR and PRAS40 reduced in the GHRKO liver, Figure 3C shows lysates prior to immunoprecipitation, which, as expected, recapitulate the findings presented in Figure 2. But that the amounts of these proteins are also reduced in mTOR complexes compared to wildtype mice.

We were unable to find a specific antibody for the mTORC2 component PROTOR1 that worked well for immunoblotting. As an alternative, we used an anti-PROTOR1 antibody for immunoprecipitation and then tested the precipitates for other mTORC2 components. We found no alteration in RICTOR, mTOR, mLST8 or mSIN1, suggesting that the level of PROTOR1 is not different between GHRKO and wildtype mice (Supplementary Figure 4). We also found a reduction of DEPTOR in the PROTOR1 immunoprecipitates, supporting our findings using RICTOR immunoprecipitation.

### DEPTOR or PRAS40 knockdown does not regulate mTORC1 or mTORC2 substrates

**Figure 4:**
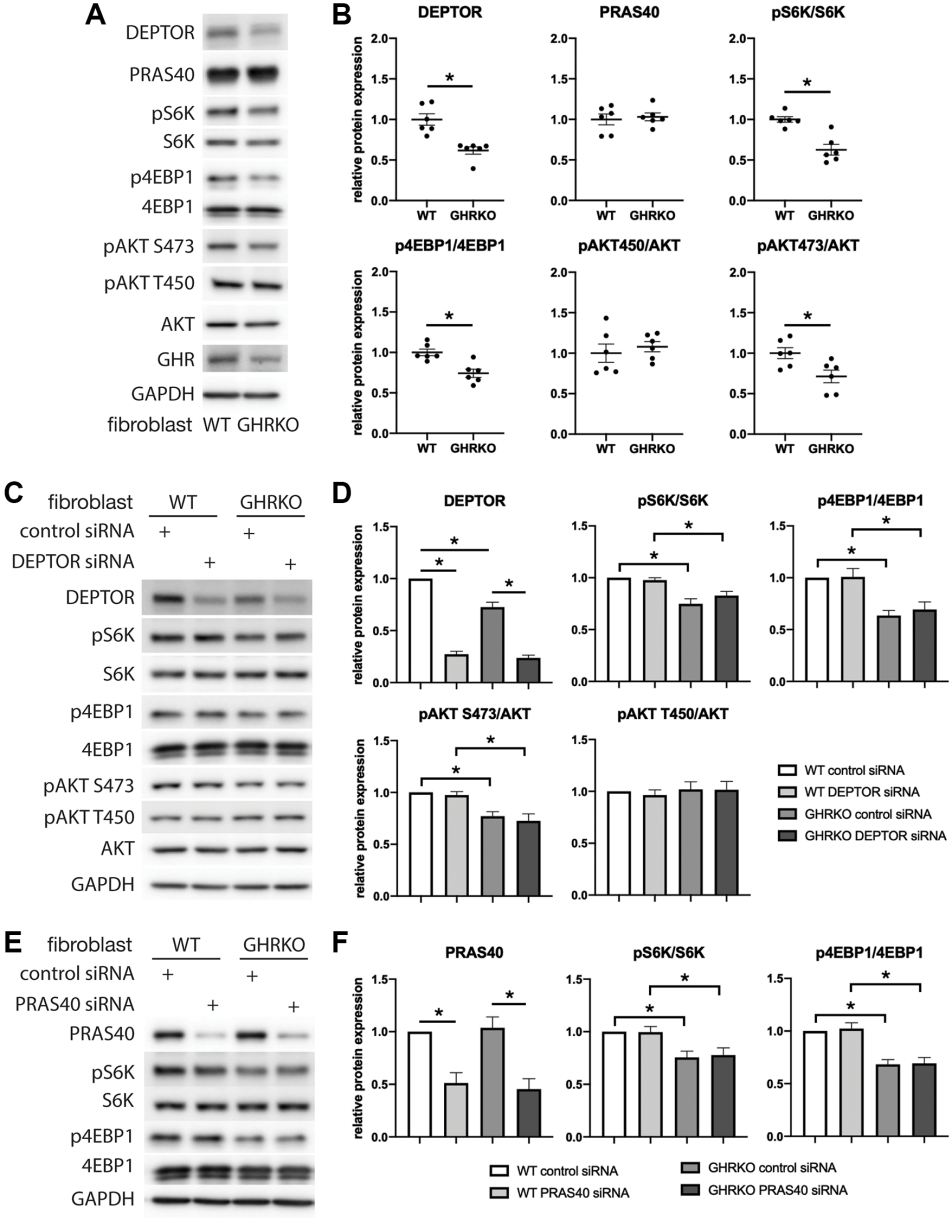
**mTORC1 or mTORC2 substrates are not regulated by DEPTOR or PRAS40 knockdown in GHRKO cells.** (**A**) Representative immunoblots of the protein expression in GHRKO cells. (**B**) Quantification of the protein levels in a series of experiment as in (**A**). (**C**, **D**) Protein expression of mTORC1 and mTORC2 substrates in DEPTOR knockdown cells. (**E**, **F**) Protein expression of mTORC1 substrates in PRAS40 knockdown cells. *N* = 6. (^*^) for *t*-test *p* < 0.05.

We next wanted to know if changes in DEPTOR and PRAS40 can regulate mTORC function in GHRKO cells, and used RNAi to knock down either DEPTOR or PRAS40 in cultures of skin-derived GHRKO fibroblasts. We also evaluated mTORC1 substrates pS6K and p4EBP1, and mTORC2 substrates pAKT S473 and pAKT T450. It has been previously reported that GHRKO mice after refeeding have lower levels of phosphorylation of mTORC1 substrates pS6K and p4EBP1, and mTORC2 substrate pAKT S473, but unchanged pAKT T450 in liver, kidney, muscle and heart [20]. We found the same changes in GHRKO fibroblasts, and a decreased expression of DEPTOR (Figure 4A, 4B). However, PRAS40 was not decreased in these fibroblasts, suggesting tissue specific regulation of this protein. Since DEPTOR is a component of both mTORC1 and mTORC2, we tested substrates of both complexes in DEPTOR knockdown cells. Surprisingly, there was no difference of the proportional phosphorylation of the mTORC substrates after DEPTOR manipulation (Figure 4C, 4D). Similarly, PRAS40 knockdown did not result in altered phosphorylation of mTORC1 substrates in GHRKO fibroblasts (Figure 4E, 4F). These results do not support the idea that changes in DEPTOR and PRAS40 lead to regulation of mTORC1 or mTORC2 function in GHRKO cells.

### Increased TSC1 and TSC2 in GHRKO mice

**Figure 5:**
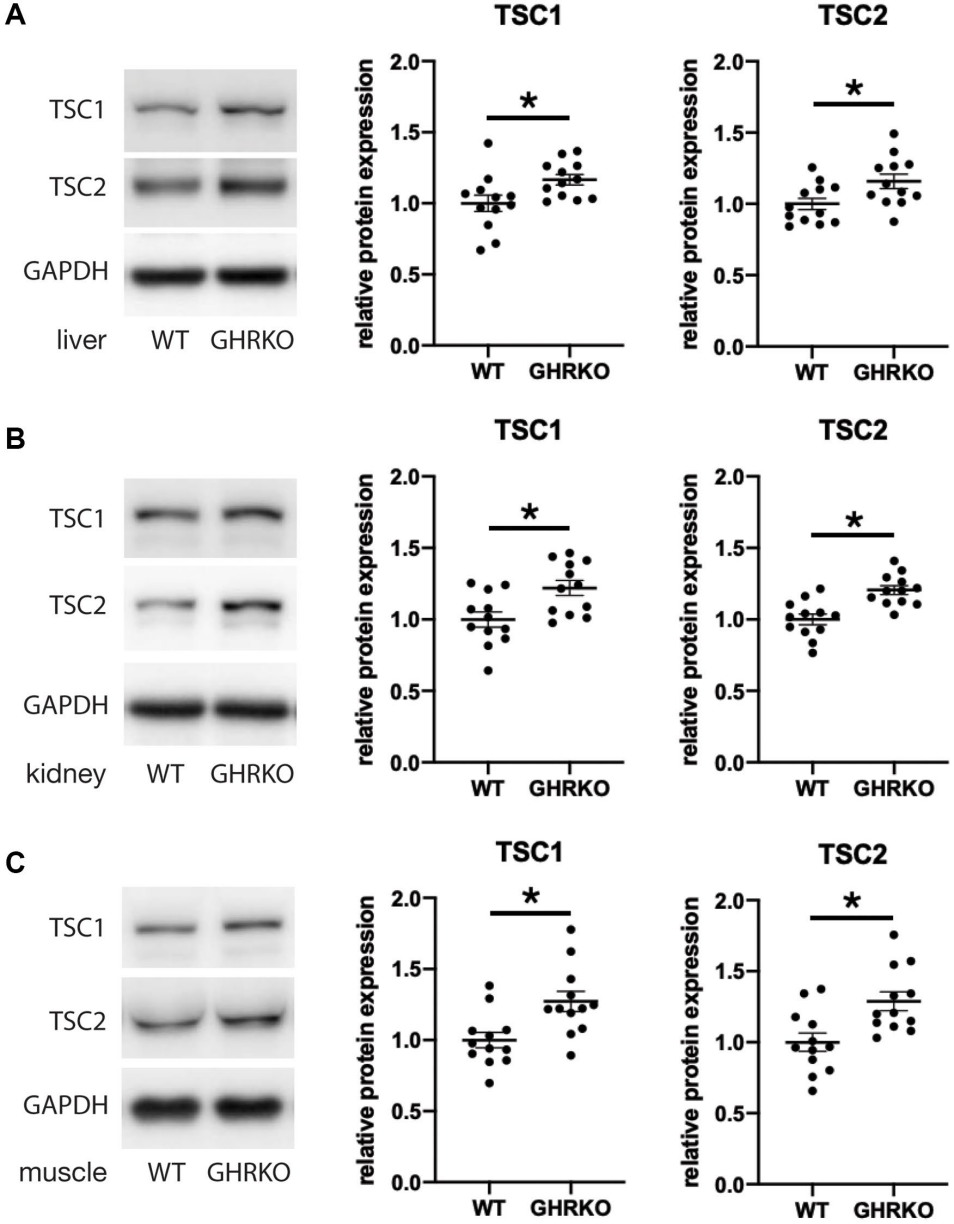
**TSC1 and TSC2 are increased in GHRKO tissues.** TSC1 and TSC2 protein expression in GHRKO liver (**A**), kidney (**B**), and muscle (**C**). *N* = 6 male and *N* = 6 female mice. ^*^t-test *p* < 0.05.

We next tested the idea that changes in the upstream regulator TSC may underlie the effects of GHRKO and Snell dwarf mutations on mTORC1 signals. We measured the protein levels of TSC1 and TSC2 in GHRKO tissues and found that both TSC1 and TSC2 are increased in GHRKO liver, kidney and muscle (Figure 5). A similar upregulation of TSC1 and TSC2 was seen in Snell dwarf liver, kidney and muscle (Supplementary Figure 5). These results show that GHRKO and Snell dwarf mice have higher levels of TSC, which could lead to inhibition of mTORC1 activity.

### Decreased phosphorylation of TSC2 in GHRKO mice

**Figure 6:**
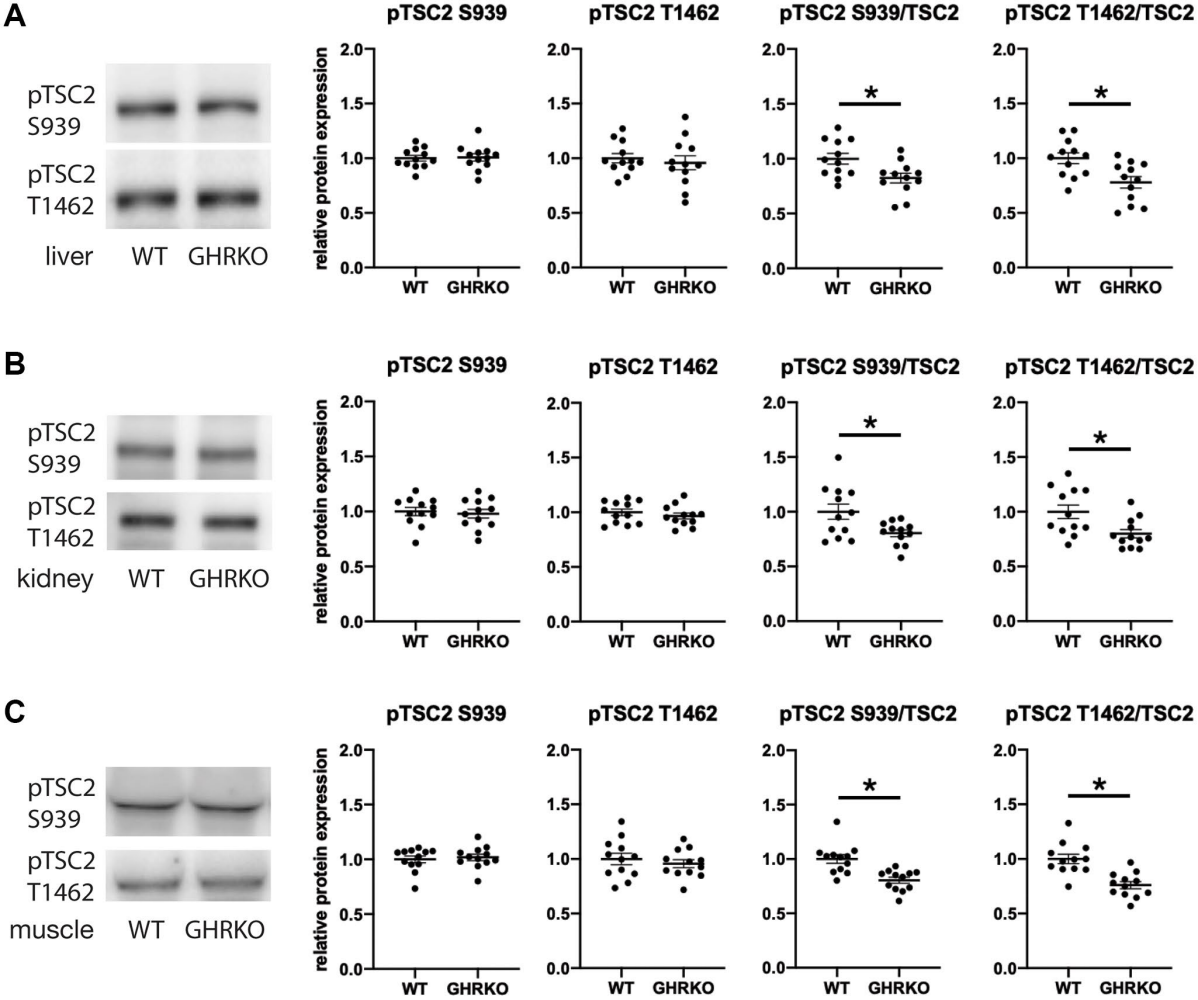
**Relative phosphorylation of TSC2 is decreased in GHRKO tissues.** The relative TSC2 phosphorylation at two sites S939 and T1462 was decreased in GHRKO liver (**A**), kidney (**B**), and muscle (**C**). *N* = 6 male and *N* = 6 female mice. ^*^t-test *p* < 0.05.

TSC2 can be phosphorylated by AKT on two sites, S939 and T1462, and in each case phosphorylation inhibits TSC activity [24, 25]. We wondered whether phosphorylation of these two sites might be modulated in GHRKO mice. We found no difference in the level of each phosphorylation form between wildtype and GHRKO liver, kidney or muscle (Figure 6). Since the total amount of TSC2 is increased in each GHRKO tissue, the ratio of phosphorylated to total TSC2 declines in each tissue, implying an increase in the amount of active, non-phosphorylated TSC2 in GHRKO mice.

### TSC2 knockdown upregulated mTORC1 activity

**Figure 7:**
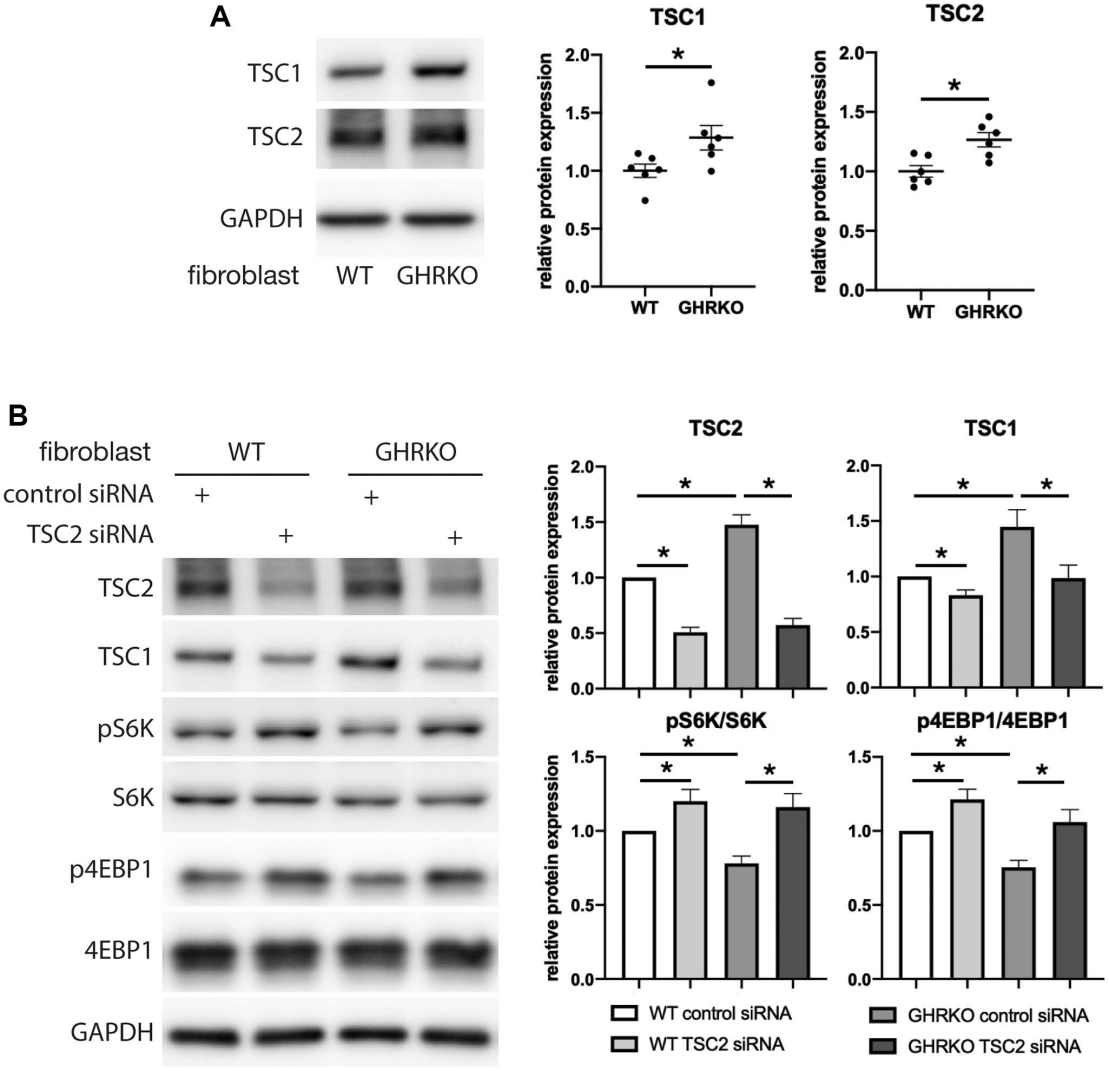
**Upregulation of mTORC1 substrates by TSC2 knockdown in GHRKO cells.** (**A**) TSC1 and TSC2 protein expression in GHRKO cells. (**B**) Protein expression of mTORC1 substrates in TSC2 knockdown cells. *N* = 6. ^*^t-test *p* < 0.05.

Because GHRKO mice have elevated TSC protein levels, we hypothesized that this causes lower mTORC1 activity in these mice. We first evaluated differences between control and GHRKO fibroblasts for TSC proteins and found, as in tissues of GHRKO mice, that both TSC1 and TSC2 are increased in GHRKO fibroblasts (Figure 7A). Then we knocked down TSC2 and found TSC1 was also decreased, suggesting coordinate regulation of these two TSC components. We tested the phosphorylation of two mTORC1 substrates S6K and 4EBP1 in these TSC2 knockdown cells (Figure 7B), and found both pS6K and p4EBP1 were augmented, suggesting that knocking down TSC2 reverses the effects of the GHRKO mutation on mTORC1 activity. These data are consistent with the idea that increased TSC1/2 in GHRKO is responsible for the decrease in mTORC1 function.

## DISCUSSION

**Figure 8:**
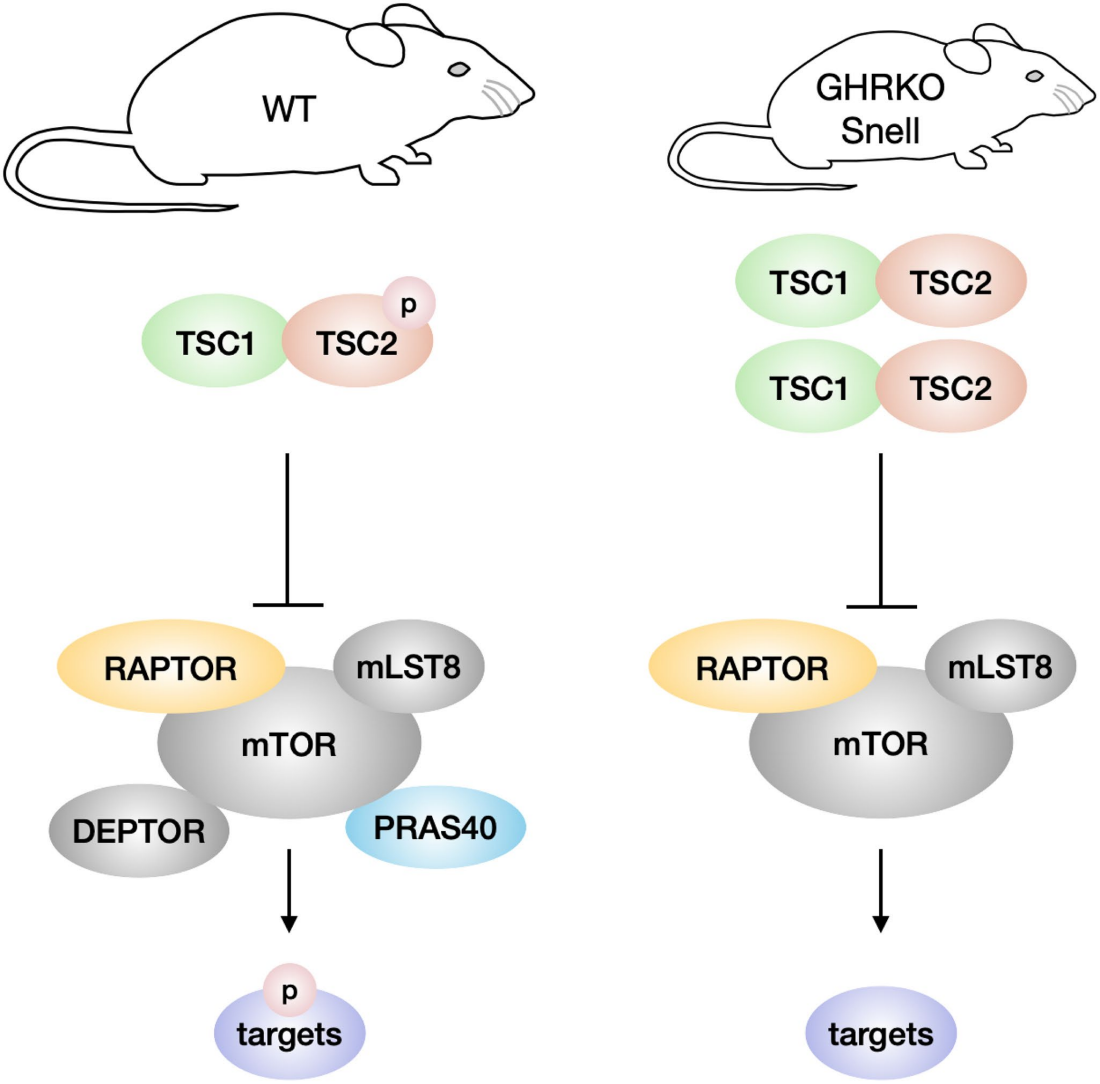
**Model of TSC and mTORC1 signaling in GHRKO/Snell dwarf mice.** In comparison with wildtype mice, GHRKO and Snell dwarf mice have higher TSC activity, shown by increased TSC1 and TSC2 protein levels, and lower TSC2 phosphorylation. Elevated TSC signaling might inhibit the mTORC1 activity of phosphorylating downstream targets. GHRKO and Snell dwarf liver have lower DEPTOR and PRAS40 levels but these changes are not responsible for the lower mTORC1 activity observed in these mice.

We have tested two ideas about the regulation of mTOR in long-lived GHRKO and Snell mice, one involving alteration in the components of the mTOR complexes, and the other focused on TSC regulation of mTORC1 function. We discovered changes in components of both mTORC1 and mTORC2, but not in the direction that could account for the previously-noted changes in mTOR activity in these mutant mice. In contrast, the change in TSC level reported here is consistent with, and might contribute to, the decline in mTORC1 function in these mutant mice (Figure 8).

We originally hypothesized that increases in DEPTOR and PRAS40 might contribute to lower levels of mTORC function, but found in contrast that these two proteins were diminished in liver of GHRKO and Snell mice. In addition, changes in these two mTORC components were not consistent among tissues, arguing against a critical role of these two proteins in regulation of mTOR activity. Furthermore, GHRKO cells with DEPTOR or PRAS40 knockdown failed to show any alteration in mTORC function. This prompted an examination of external factors, such as TSC, known to modulate mTOR function. We found an increase in total TSC1 and TSC2 with no change in the amount of phosphorylated TSC2, and thus a decline in the ratio of phosphorylated to total TSC2 levels. Since phosphorylation of TSC2 by AKT inhibits TSC activity, this finding suggests that there is more active TSC in GHRKO tissues, a change which is expected to inhibit mTORC1 activity. Moreover, TSC2 knockdown in GHRKO fibroblasts increased mTORC1 targets pS6K and p4EBP1, suggesting that TSC2 can indeed inhibit mTORC1 in these cells. Together, these results indicate that the levels and activity of TSC rather than levels of mTORC components might regulate mTORC1 activity in GHRKO and Snell dwarf mice.

In GHRKO skin-derived fibroblasts, protein expression of DEPTOR, TSC1 and TSC2, as well as mTORC1 and mTORC2 targets, is mostly consistent with results from liver, kidney and muscle, suggesting retained phenotypes in isolated cells. Our working hypothesis is that exposure to GH signals in the mouse, possibly in early post-natal life [26], leads to epigenetic switches in fibroblast cells that are retained even after they are explanted to culture. In this model, low post-natal GH signals in Ames, Snell, and GHRKO mice may be the cause of the lifespan change and increased *in vitro* stress resistance [26], as well as resistance to late-life inflammation in brain [27].

Intriguingly, DEPTOR itself can be inhibited by mTORC1 and mTORC2 at both the mRNA and protein levels [7], and we speculate that alterations of DEPTOR in these mice could reflect a feedback circuit of uncertain composition. DEPTOR can also be phosphorylated in an mTORC1-dependent manner [7]. Whether the reduction of DEPTOR in GHRKO and Snell liver is caused by mTORC2 and whether DEPTOR phosphorylation affects its levels would be worth investigating. PRAS40 can be phosphorylated by AKT and mTORC1, releasing the inhibitory effects on mTORC1 [28, 29]. Whether these phosphorylation events affect the level of PRAS40 in GHRKO and Snell dwarf mice awaits further testing.

In addition to DEPTOR and PRAS40, other mTORC components can also be regulated post-translationally. The core mTOR kinase can be phosphorylated at S2448 by S6K in response to serum stimulation [30]. mTOR autophosphorylates at S2481, an indicator of intact mTORC2 assembly [31]. In response to energy stress, the mTORC1 scaffold protein RAPTOR is phosphorylated by AMPK at S722 and S792 to inhibit mTORC1 function [32]. Interestingly, the mTORC2 scaffold protein RICTOR is phosphorylated by the mTORC1 substrate S6K at T1135 to inhibit mTORC2 substrate phosphorylation of AKT at S473, adding additional complexity to the mutual regulation between mTORC1 and mTORC2 [33]. Moreover, mSIN1 can be phosphorylated by AKT at T86 to further activate mTORC2, leading to phosphorylation of AKT at S473 [34]. These phosphorylation events display integrated signaling of mTORC1 and mTORC2 regulation with sophisticated feedback mechanisms. It would be necessary to study how these phosphorylation events of mTORC components change in GHRKO and Snell dwarf mice and how they might influence mTORC activity and substrate specificity.

As an upstream regulator of mTORC1 activity, manipulation of TSC might lead to phenotypes similar to those seen in slow-aging mice. Overexpression of the *Drosophila* orthologs of TSC1 and TSC2, dTSC1 or dTSC2, in fruit flies leads to lifespan extension. Similar effects are seen in *Drosophila* bearing mutations in dTOR and dS6K [3]. Moreover, TSC1 overexpression in mice improves their exercise performance and heart function, and extends the lifespan of female but not male mice [35]. It would be interesting to know whether overexpression of TSC2 has similar effects of increased healthspan and lifespan in mice. In addition to the two phosphorylation sites of TSC2 tested here, there are several other residues on TSC2 and TSC1 phosphorylated by kinases such as AMPK, ERK and CDK1 to transit upstream signals to mTORC1 [36]. Whether these phosphorylation sites are also present and functioning in GHRKO and Snell dwarf mice would be worth following up.

In summary, this study highlights the potential role of TSC in regulating mTORC1 activity in long-lived mutant mice. Given the fact that deficient mTORC1 signaling extends lifespan in different species including mice, the regulation of mTOR complexes may be one of the reasons for extended lifespan of GHRKO and Snell dwarf mice. Inhibiting TSC in these mice would be helpful to know whether TSC inhibits mTORC1 activity *in vivo*, and whether reversing mTORC1 activity also abolishes the lifespan extension of GHRKO and Snell dwarf mice. Future studies on enhancing TSC function by overexpressing TSC2 or using TSC2 activating molecules may generate additional mouse models that can be used as comparisons with GHRKO and Snell dwarf mice, which would provide more evidence about the negative role of mTOR in aging.

## METHODS

### Mice

All animal experiments were conducted with approval of the Institutional Animal Care and Use Committee at University of Michigan. Mice were housed in same sex cages in a specific pathogen-free colony, given free access to mouse chow and water, and euthanized in the ad libitum-fed condition at the same time of the day. GHRKO mice were generated by deleting partial growth hormone receptor gene around the fourth exon as described in [18]. Snell dwarf mice were bred by crossing heterozygous male and female mice with the Pit1 gene mutation as described in [19]. Six-month old control and mutant animals were used for experiments.

### Cell culture and RNAi

Fibroblasts were isolated from GHRKO mouse tail tips using collagenase (Gibco, 17101–015) and cultured in DMEM (Gibco, 11965092) with 10% fetal bovine serum (Corning, 35011CV) and 1% antibiotic-antimycotic (Gibco, 15240062). Cells were cultured in a 37°C incubator with 10% CO_2_ and were used at passages 3 or 4. RNAi was done using Opti-MEM (Invitrogen 31985062) and lipofectamine (Invitrogen 13778150) according to the manufacturer’s instructions. The following siRNAs were used, control (Invitrogen 4390844), DEPTOR (Invitrogen n424206), PRAS40 (Invitrogen s85345), TSC2 (Invitrogen s75509). Cells were harvested for immunoblot analysis after two days of transfection.

### Immunoblot

Proteins were extracted from tissues or cells using a lysis buffer containing 33 mM Tris-HCl pH 6.8, 5% glycerol and 1% SDS. Protein concentration was measured by BCA assay (Thermofisher 23227). 50 μg tissue protein or 20 μg cell protein were loaded into SDS-PAGE gels, transferred onto PVDF membranes and incubated by the antibodies listed in (Supplementary Table 1) at 4°C overnight. The blots were developed using ECL prime reagent (Cytiva RPN2232). Blot images were quantified using ImageJ. Quantification of the bands were normalized to GAPDH, and the average of controls was set as 1. Phosphorylation levels were compared directly to total levels of the same protein, and then expressed relative to levels in controls.

### Immunoprecipitation

20 mg portions of tissues were lysed in 600 μL lysis buffer with HEPES 40 mM pH 7.5, NaCl 120 mM, EDTA 1 mM, Na-pyrophosphate 10 mM, glycerophosphate 10 mM, NaF 50 mM and 0.3 % CHAPS. 2 μg antibody was mixed with pre-washed protein A magnetic beads (Biorad 1614013) and rotated at 4°C for 2 hrs. 300 μL tissue lysates were incubated with the antibody at 4°C overnight. Proteins were eluted with 40 μL 1X Laemmli buffer (Biorad 1610747) at 70°C for 10 min.

### Statistics

Immunoblot results were analyzed by two-way ANOVA (Sex × Genotype, with Interaction), to see if the genotype effects were sex specific. Since none of the interaction terms were statistically significant, data were then combined from both males and females and significance assessed using the Student’s *t*-test.

### Data availability statement

All original data are available upon request.

## Supplementary Materials

Supplementary Figures

Supplementary Table 1
