# Supplementary material for: Regulation of mTOR complexes in long-lived growth hormone receptor knockout and Snell dwarf mice
Source: Aging (Albany NY). 2022 Mar 19;14(6):2442–61. doi: 10.18632/aging.203959 (PMC9004569; doi:10.18632/aging.203959)
Supplement: Supplementary Figures [file aging-14-203959-s001.pdf]

SUPPLEMENTARY FIGURES

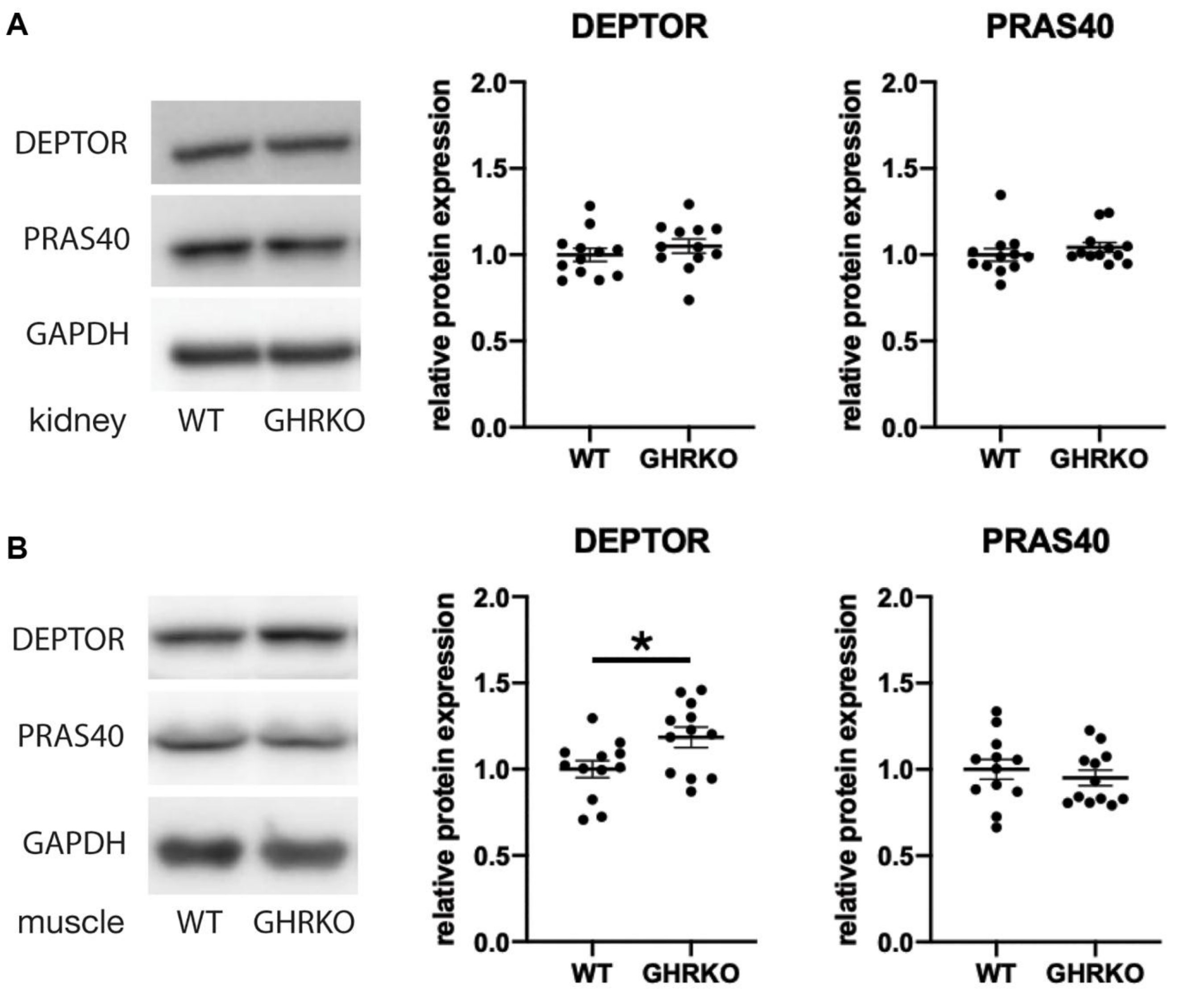

Supplementary Figure 1. DEPTOR and PRAS40 are not reduced in GHRKO kidney and muscle. (**A**) Protein expression of DEPTOR and PRAS40 in GHRKO kidney. (**B**) Protein expression of DEPTOR and PRAS40 in GHRKO muscle. *N* = 6 male and *N* = 6 female mice. *t-test $p < 0.05$.

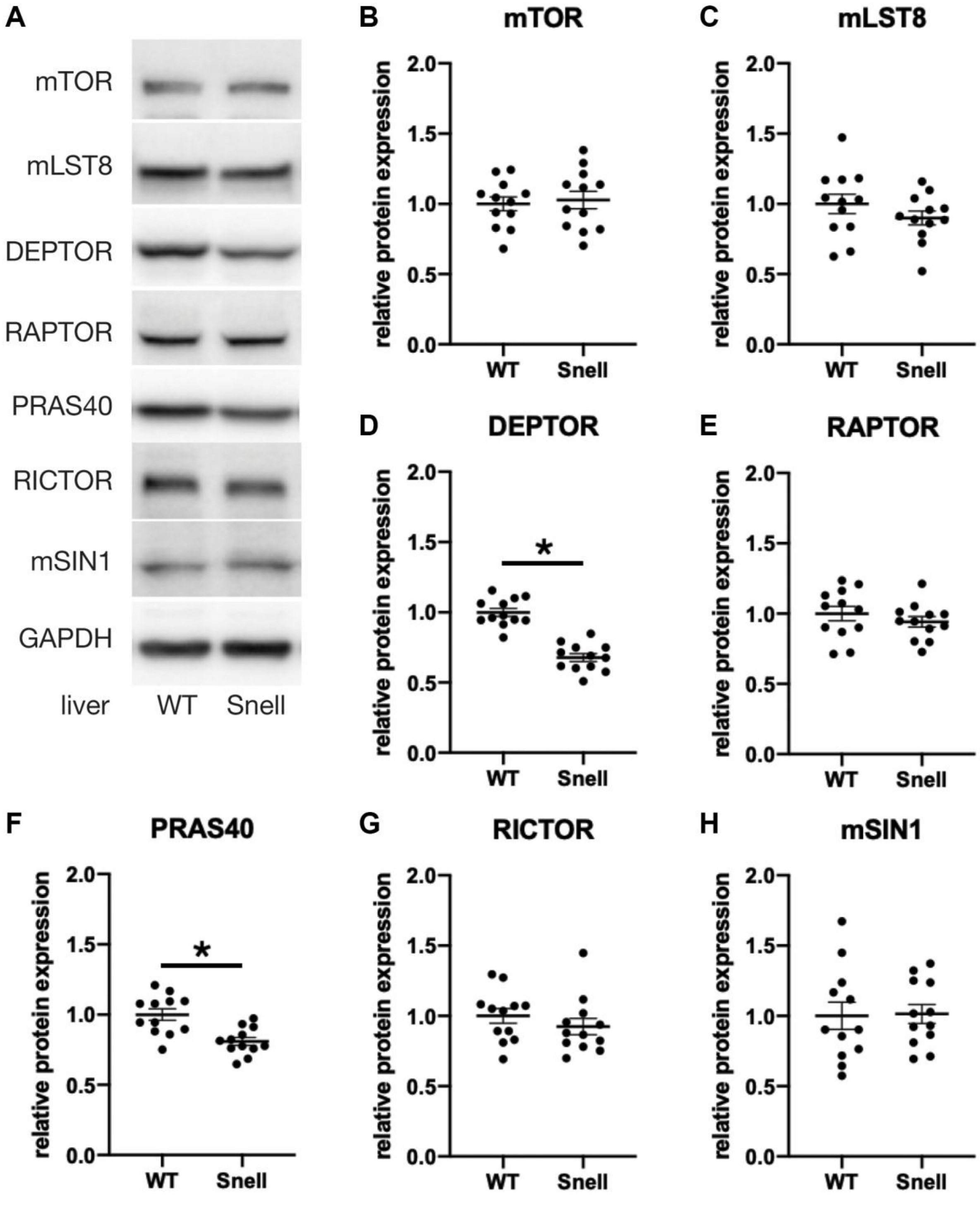

Supplementary Figure 2. Reduced DEPTOR and PRAS40 protein expression in Snell dwarf liver. (**A**) Representative immunoblots of protein expression for mTORC1 and mTORC2 components. (**B**–**H**) Quantification of protein expression, for *N* = 6 male and *N* = 6 female mice, with mean and SEM. $^*t$-test $p < 0.05$.

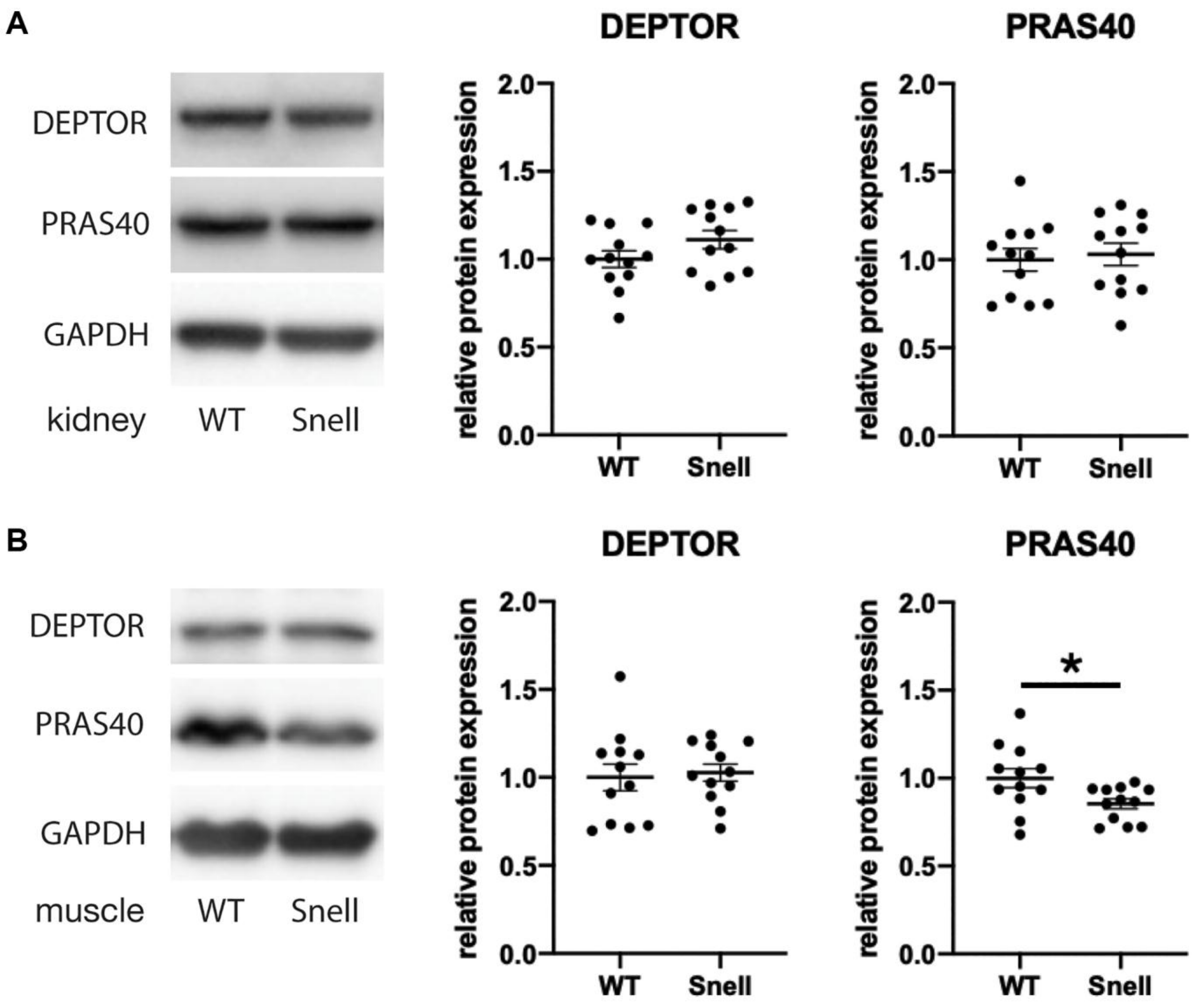

Supplementary Figure 3. PRAS40 is reduced in Snell dwarf muscle. (**A**) Protein expression of DEPTOR and PRAS40 in Snell dwarf kidney. (**B**) Protein expression of DEPTOR and PRAS40 in Snell dwarf muscle. N = 6 male and N = 6 female mice. $^*t$-test $p < 0.05$.

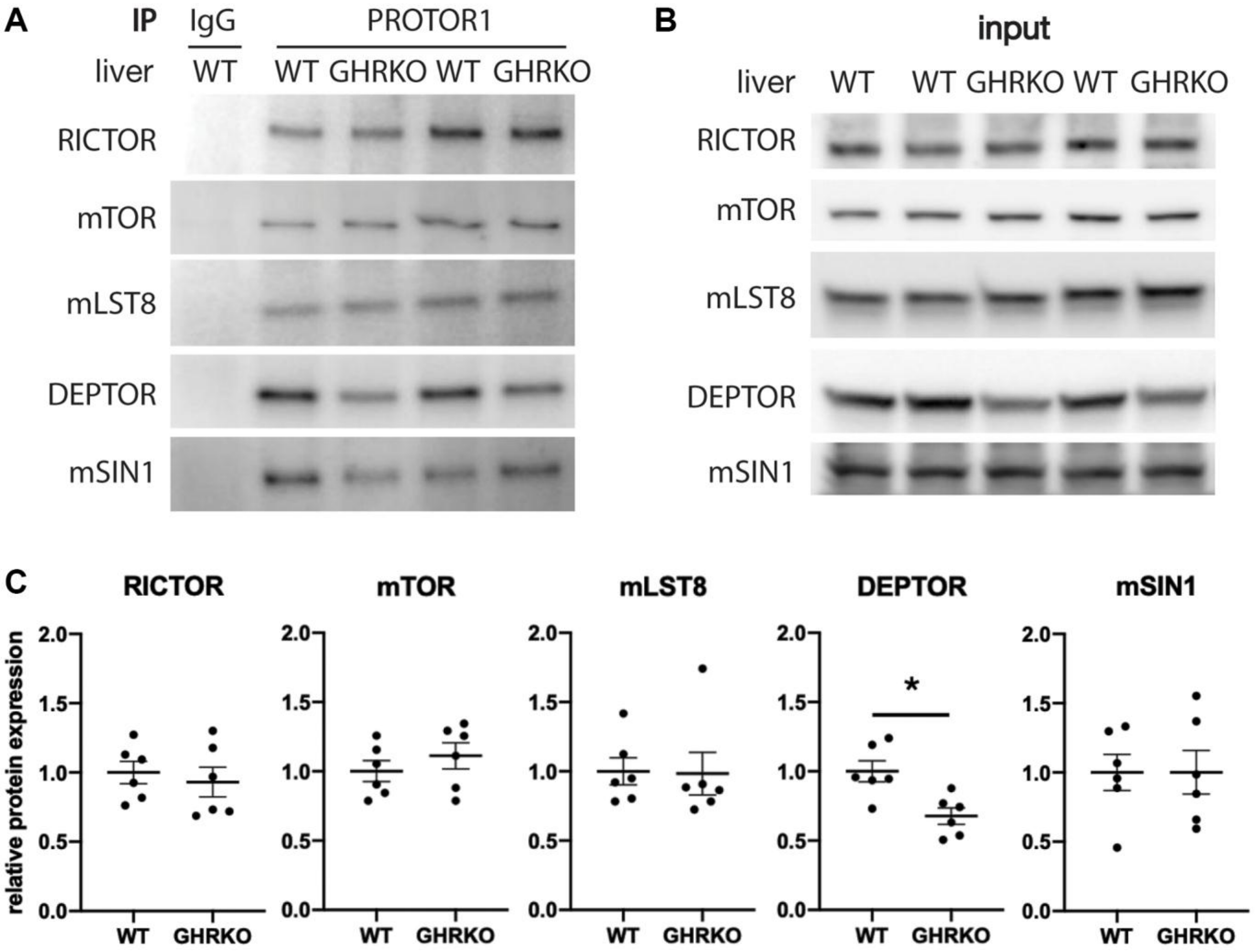

Supplementary Figure 4. Unchanged PROTOR1 level in mTORC2. (**A**) Representative immunoblots of the mTORC2 components from samples immunoprecipitated with PROTOR1 antibody. (**B**) Input samples for (**A**). (**C**) Quantification of proteins in experiments of the kind shown in (**A**). $N = 6$. $^{*}t$-test $p < 0.05$.

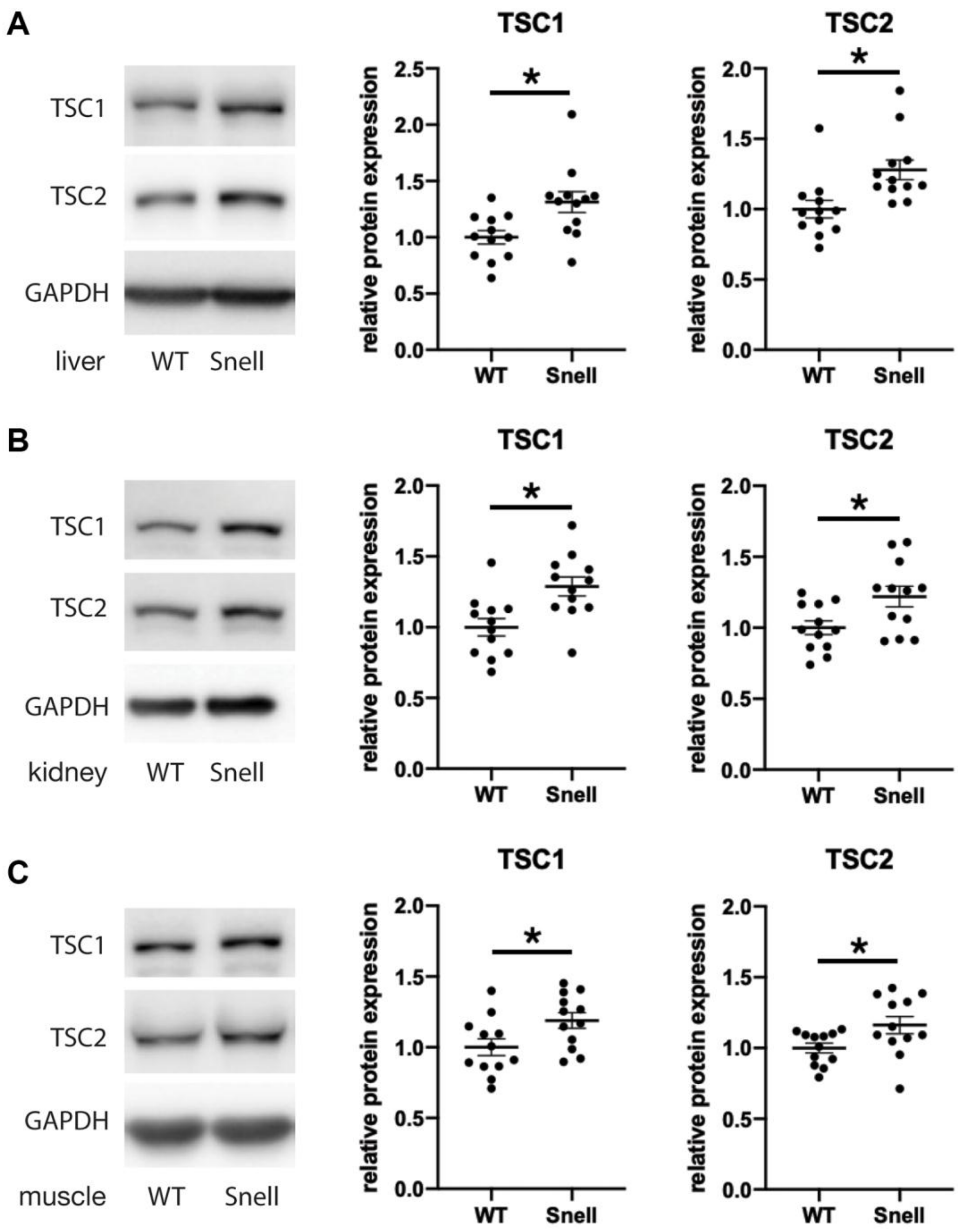

Supplementary Figure 5. TSC1 and TSC2 are increased in Snell dwarf tissues. TSC1 and TSC2 protein expression in Snell dwarf liver (**A**), kidney (**B**), and muscle (**C**). *N* = 6 male and *N* = 6 female mice. $^{*}t$-test $p < 0.05$.
