# Supplementary material for: Regulation of mTOR complexes in long-lived growth hormone receptor knockout and Snell dwarf mice
Source: Aging (Albany NY). 2022 Mar 19;14(6):2442–61. doi: 10.18632/aging.203959 (PMC9004569; doi:10.18632/aging.203959)
Supplement: Supplementary Table 1 [file aging-14-203959-s002.pdf]

SUPPLEMENTARY TABLE

Supplementary Table 1. List of antibodies.

Name	Source	Catalog
GAPDH	Cell Signal	2118S
mTOR	Cell Signal	2972S
mLST8	Cell Signal	3274S
DEPTOR	Novus Bio	NBP1-49674
RAPTOR	Thermofisher	42–4000
PRAS40	Cell Signal	2691S
RICTOR	Abcam	ab70374
mSIN1	Abcam	ab64188
PROTOR1	Bethyl lab	A304–187A
TSC1	Cell Signal	6935S
TSC2	Cell Signal	4308S
pTSC2 S939	Cell Signal	3615S
pTSC2 T1462	Cell Signal	3617S
p4EBP1	Cell Signal	2855S
4EBP1	Cell Signal	9644S
pS6K	Cell Signal	9234S
S6K	Cell Signal	9202S
pAKT S473	Cell Signal	4060S
pAKT T450	Cell Signal	9267S
AKT	Cell Signal	9272S
GHR	R&D Systems	AF1360
rabbit IgG	Cell Signal	2729S
anti-rabbit	Abcam	ab205718
anti-goat	R&D Systems	HAF017
